# Molecular tools to create new strains for mosquito sexing and vector control

**DOI:** 10.1186/s13071-018-3209-6

**Published:** 2018-12-24

**Authors:** Irina Häcker, Marc F. Schetelig

**Affiliations:** Institute for Insect Biotechnology, Justus-Liebig-University Giessen, Heinrich-Buff-Ring 26-32, 35392 Giessen, Germany

**Keywords:** mosquitoes, vector control, sexing systems, genome modification, transposable elements, site-specific recombination, genome editing, CRISPR/Cas, TALEN

## Abstract

Vector control programs based on population reduction by matings with mass-released sterile insects require the release of only male mosquitoes, as the release of females, even if sterile, would increase the number of biting and potentially disease-transmitting individuals. While small-scale releases demonstrated the applicability of sterile males releases to control the yellow fever mosquito *Aedes aegypti,* large-scale programs for mosquitoes are currently prevented by the lack of efficient sexing systems in any of the vector species.

Different approaches of sexing are pursued, including classical genetic and mechanical methods of sex separation. Another strategy is the development of transgenic sexing systems. Such systems already exist in other insect pests. Genome modification tools could be used to apply similar strategies to mosquitoes. Three major tools to modify mosquito genomes are currently used: transposable elements, site-specific recombination systems, and genome editing via TALEN or CRISPR/Cas. All three can serve the purpose of developing sexing systems and vector control strains in mosquitoes in two ways: first, via their use in basic research. A better understanding of mosquito biology, including the sex-determining pathways and the involved genes can greatly facilitate the development of sexing strains. Moreover, basic research can help to identify other regulatory elements and genes potentially useful for the construction of transgenic sexing systems. Second, these genome modification tools can be used to apply the gained knowledge to build and test mosquito sexing strains for vector control.

## Background

The control of pest insects via the sterile Insect technique (SIT) has been performed successfully for several decades. The most prominent examples are the eradication of the tsetse fly *Glossina austeni* in Zanzibar [1], of the screwworm *Cochliomyia hominivorax* from Mexico, the US, and Central America [2], and the successful suppressive or preventive control programs for the Mediterranean fruit fly *Ceratitis capitata* in North and Central America [3–7]. The SIT offers a highly species-specific and therefore environment-friendly approach for insect pest control. The SIT is based on the mass release of males of the target species sterilized by irradiation. Matings of these sterile males with wild type females in the field will not produce offspring, thereby decreasing the population size of the next generation. Via repeated releases, the population can be reduced to a manageable size. The release of only male insects is beneficial for the SIT as well as similar control strategies based on male sterility. It increases the efficacy of the program and thereby reduces the costs [8]. First trials to establish control programs based on transgenic sterility have also been implemented for the yellow fever mosquito *Aedes aegypti*. Several small-scale releases were performed with a self-limiting strain of *Ae. aegypti* in Grand Cayman [9, 10] and Brazil [11, 12]. The strain carries a transgenic construct that kills most of the offspring of the released males during late larval or pupal development [13]. All releases showed a significant reduction of the *Ae*. *aegypti* populations in the release areas.

While male-only releases are desirable for agricultural pests, they are a prerequisite for all control programs involving the mass release of insect vectors. In insect vectors, only the females bite and can thereby transmit diseases. Thus, the release of females, even if sterile, would increase the number of biting and potentially disease-transmitting individuals. Elimination of female mosquitoes for the small-scale releases of *Ae. aegypti* (0.5 to 1.5 million males per week) was performed mechanically, making use of the size difference between male and female pupae for separation. Mechanical sexing as it is currently performed, however, is not only labor intensive, time consuming and costly. It is also not 100% efficient, with a female contamination of 0.02% or more [11, 14]. Moreover, this strategy is not applicable to anophelines as the pupal size difference is mostly not pronounced enough for successful separation [15]. Therefore, efficient sexing systems are urgently needed for the major vector species. Only then large-scale control programs based on the release of sterile males, where up to one billion males per week are produced, can be developed. Such sexing systems would be desirable for *Ae. aegypti*, *Ae*. *albopictus*, and *Ae*. *polynesiensis*, which are the major vectors of yellow fever, dengue, chikungunya and Zika virus, and for the malaria vectors of the *Anopheles gambiae* complex and *An. stephensi.*

Several approaches exist besides mechanical or physical sex separation in mosquitoes [16]. One strategy is classical genetic mutagenesis using chemicals or irradiation and screening for the desired phenotype. An example of such a genetic sexing strain (GSS) in mosquitoes is the *An. arabiensis*, ANO IPCL1 strain, where a mutation confers dieldrin resistance to males, and kills the susceptible females when eggs or larvae are treated with the insecticide [17]. GSS also exist for *An. gambiae* [18] and *An. albimanus* [19]. The development of a GSS using classical genetic approaches can take a long time, however, and cannot be easily transferred to another species, as the induction of mutations via chemicals or irradiation results in random mutagenesis. Therefore, the underlying molecular basis of the mutant phenotype is often not known.

Transgenic strategies offer multiple approaches to create sexing systems in mosquitoes. Besides their usefulness in the construction of sexing strains, transgenic technologies have become an important factor in basic research, e.g. by using transposable elements to uncover gene function in insertional mutagenesis studies or to identify regulatory elements in enhancer trap experiments. Furthermore, genome editing technologies have recently been used to help elucidate the sex-determining pathway in *Ae. aegypti* [20]. This basic research on mosquito biology creates a strong basis for the development of sexing strains, as it uncovers potential candidate genes and regulatory elements that can be used to construct a transgenic sexing system (TSS). TSSs already exist in several insect pests, relying on different strategies. In *Anopheles* and *Aedes* mosquitoes, fluorescent protein marker expression was linked to the sex. Mechanical sorting of the male from female larvae was then achieved based on the absence or presence of fluorescence by using a flow cytometer, the Complex Object Parametric Analyzer and Sorter (COPAS) [21–23]. In a different approach, transgenic embryonic sexing systems (TESS) were built for several agricultural pests like the Mediterranean fruit fly *Ceratitis capitata* [24], the Caribbean fruit fly *Anastrepha suspensa* [25], and the Australian Sheep Blow fly *Lucilia cuprina* [26]. They are based on the conditional elimination of female insects during early embryonic development to obtain only male insects for release. Genome modification tools offer the possibility to transfer such successful sexing strategies to important vectors and adapt them for each species. Three major tools to modify mosquito genomes are available at the moment and are functional in four important vectors from the genera *Aedes* and *Anopheles*: transposable elements, site-specific recombination systems, and genome editing via TALEN or CRISPR/Cas. Here, we review these three major technologies for genome modification in insects, specifically focusing on their application and achievements in mosquitoes with respect to their potential to create sexing strains for vector control.

## Germline transformation of mosquitoes using class II transposable elements

Transposons are mobile genetic elements found in almost all life forms and can make up a large fraction of an organism’s genome. The first transposon used to transform an insect genome was the P element in *Drosophila melanogaster* in the 1980s [27]. Expectations to have found a universal tool for insect transgenesis were not confirmed, however, when researchers essentially failed to apply it to other insects. The positive effect of this failure was an intense search for other transposons suitable for germline transformation of insects since the late 1990s. This led to the discovery of nine different transposable elements, *Hermes, Herves, Hobo, Hopper, Minos*, Mos1, the *P* element, *piggyBac*, and *Tn5*.

Four of them are regularly used for insect transformation: the hAT-related element *Hermes* from the housefly *Musca domestica*, the *Minos* element from *Drosophila hydei*, the *mariner* family transposon Mos1 from *D. mauritiana*, and the *piggyBac* transposon from the cabbage looper *Trichoplusia ni*. With their help, many different insects have been transformed, including lepidopteran, dipteran, and coleopteran species. Due to its precise cut-and paste mechanism and high mobility in many insect genomes, *piggyBac* has become the most widely and most frequently used transposable element with more than 30 different species on the transformation list. All four transposons have also been successfully applied for mosquito transformation (Table 1).

**Table 1:** Transposon vector systems used in mosquitoes

Transposon	Species	First publication
*Hermes*	*Aedes aegypti*	Jasinskiene et al. 1998 [151]
	*Culex quinquefasciatus*	Allen et al. 2001 [152]
*Minos*	*Anopheles stephensi*	Catteruccia et al. 2000 [153]
*Mos1/mariner*	*Aedes aegypti*	Coates et al. 1998 [154]
*piggyBac*	*Aedes aegypti*	Kokoza et al. 2001 [28]
	*Anopheles gambiae*	Grossman et al. 2001 [155]
	*Anopheles stephensi*	Nolan et al. 2002 [156]
	*Anopheles albimanus*	Perera et al. 2002 [157]
	*Aedes fluviatilis*	Rodrigues et al. 2006 [158]
	*Aedes albopictus*	Labbe et al. 2010 [80]
*TN5*	*Aedes aegypti*	Rowan et al. 2004 [159]

### Mobility of transposable elements in the mosquito genome

The mobility of transposable elements is an important feature to uncover the function of genetic elements in enhancer-trapping and gene-tagging experiments as well as in genome-wide mutagenesis studies. In the *Ae. aegypti* genome, however, transposable elements show a very limited mobility or are completely immobile. Although the *piggyBac* transposase in *Aedes* integrates in a precise transposition event, leading to the canonical duplication of the TTAA recognition sequence [28], it does not remobilize [29, 30]. The results obtained from a variety of experiments indicates the involvement of species-specific effects in *Ae. aegypti*. While *piggyBac* remobilizes in plasmid-based assays in the *Ae. aegypti* cell line Aag2, it is immobile when it is stably integrated in that cell line [29]. Esnault et al. discovered that local context effects involving the surrounding 500-1000 bp of the genomic integration site influence the mobility of the *piggyBac* transposon in *D. melanogaster* [31]. This led to the reasoning that such context effects might also be responsible for the immobility of *piggyBac* in *Aedes*. However, a *piggyBac* transposon transferred from the *Ae. aegypti* genome into *D. melanogaster* together with the flanking 1000 bp of the *Ae. aegypti* genomic integration site remobilizes with high efficiency in the *D. melanogaster* genome [29]. This result contradicts the local context hypothesis. As the *piggyBac* construct and flanking sequences were isolated from the *Ae. aegypti* genome by PCR, essentially blank DNA (lacking all chromatin information) was transformed into *D. melanogaster*. Therefore, the more likely candidates for the silencing effect of transposable elements in *Ae. aegypti* would be genetic or epigenetic effects.

To our knowledge, the mobility of genetic elements has not been thoroughly tested in any other mosquito of the genus *Aedes*. The only other experiment addressing *piggyBac* mobility in *Aedes* is based on inter plasmid assays in *Ae. albopictus* embryos [32], where the element was mobile. However, taking into account the results in *Ae. aegypti*, inter plasmid assays do not necessarily allow conclusions on the behavior of genomic *piggyBac* integrations.

Germline remobilization experiments with other insect class II transposons such as *Hermes* and *Mos1* display similar behavior within the *Ae*. *aegypti* germline. *Hermes* shows a very limited remobilization in *Ae. aegypti* under different experimental conditions. This includes the embryonic microinjection of a helper plasmid encoding the transposase under the control of the *D*. *melanogaster hsp70* promoter [33], as well as crossing the transgenic line carrying the *Hermes* construct to a jumpstarter line [34]. The jumpstarter line permanently expresses the transposase in the testis. Also, the remobilization of a *Mos1*-based gene vector in *Ae. aegypti* was extremely rare (1 in 14,000 G_1_) [35]. The reduced *Hermes* mobility seemed to be an *Aedes*-specific phenomenon, as it remobilizes within the *D. melanogaster* germline at a rate of approximately 0.03 jumps per element per generation [36]. In contrast, low *Mos1* mobility was also observed in *D. melanogaster* with a remobilization rate of less than 1% [37].

Within other mosquito genera, the inability of transposons to remobilize has been observed for the *Minos* element in *Anopheles stephensi.* Here, germline remobilization assays did not yield any positive events in more than 35,000 screened G_1_, although evidence of somatic mobility was recovered. These somatic events, however, occurred to a large extent with a non-canonical mechanism [38].

In contrast to the general immobility of class II transposable elements in *Ae. aegypti*, and of *Minos* in *An. stephensi*, the *piggyBac* transposon vector system is highly mobile in *An. stephensi* and *An. gambiae,* where it is exploited as a tool for enhancer trap studies [39, 40].

### Hyperactive versions of transposases

Transformation efficiency with transposable elements in general is low, reaching rarely more than 10 to 15% and being often much lower. Their broad applicability in insects and mammalian cells, nevertheless turns them into important tools for transgenesis. It would therefore be desirable to increase transposition efficiency. This lead to different efforts to enhance the activity of transposases, starting by codon-optimizing the insect *piggyBac* transposase for mammalian usage [41, 42].

By screening for hyperactive mutants of the insect *piggyBac* transposase in yeast, a hyperactive version (IPB7) was created. To further increase expression of the transposase for use in mammalian cells, the combination of the best hyperactive mutations was transferred into the mammalian codon-optimized enzyme, which was named hyPBase [43]. Wright et al. tested the insect codon mutant IPB7 in *Ae. aegypti*. Despite a 9-fold increased activity of the mutant in mammalian cells [43], IPB7 had no effect in *Ae. aegypti* inter-plasmid assays compared to the wild type *T. ni piggyBac* transposase [44]. Moreover, the enzyme caused a high degree of sterility in transformed individuals, that was also observed in a parallel experiment in *D. melanogaster*. Investigation of sterile *Drosophila* and *Aedes* females showed that sterility was caused by a severe atrophy in the ovaries of females injected with the mutant helper plasmid alone or in combination with a donor [44]. Interestingly, the same hyPBase tested again in *D. melanogaster, C. capitata,* and *T. castaneum,* did not cause such high sterility rates [45]. Thus, hyperactive *piggyBac* versions could be evaluated again for effective genetic engineering and enhanced mosquito transformation.

Additionally, mutated versions of *Mos1* were tested in *Ae. aegypti*. These mutants were based on the mutations generated in a related transposon *Himar1* [46]. In combination with perfectly matched 5’ and 3’ ITRs, two of the *Mos1* mutants showed a more than 3-fold increase in transposition activity, while at the same time significantly improving the integrity of the transposition process [47].

### Advantages and disadvantages of mosquito transgenesis with transposons

Commonly used transposable elements use short genomic recognition sequences for integration, such as TA for *mariner* or TTAA for the *piggyBac* transposase. Due to the high frequency of such short recognition sequences in the genome, the integration is essentially random, which can lead to insertional mutagenesis effects. Scientists make use of the random integration to uncover new regulatory elements within genomic regions and to identify gene function in enhancer trap and insertional mutagenesis experiments, respectively. For this purpose, scientists favor transposons with strong remobilization properties to be able to hit many different genomic locations in one experiment. Due to the nearly complete immobility of transposable elements in the *Ae. aegypti* genome, this important tool for genome wide functional studies is not available in the yellow fever mosquito. In contrast, it has been successfully applied to the Asian and African malaria mosquito [39, 40].

If the purpose of the transformation is the creation of transgenic mosquitoes, e.g. sexing strains, then insertional mutagenesis is an unwanted side effect, as it can be deleterious for the fitness of the transgenic insects. Depending on the affected gene, the consequences may range from mild phenotypic effects to lethality [48]. Extensive fitness tests are necessary to assess the biological quality and mating competitiveness of transgenic strains first at a laboratory scale, and then in (semi-) field cages and in a mass rearing setup and small-scale releases. In some cases, the created transgenic strains do not show obvious defects in biological quality under normal lab rearing conditions [48, 49]. It has been shown, however, that small negative effects on strain fitness based on the transgene integration can remain hidden under optimal rearing conditions, but become measurable when the strains are put under stress, such as limited food resources [50]. Thus, even if transgenic strains show suitable fitness in the lab, they might not perform well enough under mass rearing conditions, which can involve a certain level of stress, such as density stress and limited food resources. Moreover, fitness loads might only be noticeable in the homozygous state, while they remain obscured as long as one intact allele is present (compare [51, 52] and [48, 49, 53]). Besides these direct effects of insertional mutagenesis, an indirect fitness effect can result from deleterious genes located close the integration site. Due to selection for the transgene in each generation and low frequency of recombination between the transgene locus and a deleterious gene located nearby, the latter enriches in the population, thus decreasing fitness. Together, these effects will affect the suitability of the strains for pest control applications.

The random integration into the genome can also lead to genomic position effects caused by regulatory elements like enhancers and silencers, or to position effect variegation due to nearby heterochromatic regions. There are several examples where the same transgene construct integrated at different genomic positions shows varying degrees of expression [54–58]. Therefore, it is impossible to perform an unbiased comparison of the function and efficacy of different transgene constructs located at different genomic positions.

With the overall low transformation frequency achieved with transposable elements, it can be time-consuming to obtain a transgenic strain with the appropriate transgenic characteristics and sufficient biological quality.

### Use of transposable elements to create sexing strains in mosquitoes

Fluorescent proteins are commonly used in insects as markers to identify transgenic individuals. The male- (or sex-) specific expression of fluorescent markers can be exploited for sex separation. This strategy has been pursued for *Aedes* and *Anopheles* species. Sex separation is e.g. achieved by mechanical sorting with the COPAS. Using the *beta2-tubulin (β2-tub*) promoter to express the fluorescent marker protein allows reliable sorting for marked males from non-marked females in *Ae. aegypti* [23] and *Anopheles stephensi* [21]. Using the *An. gambiae dsx* promoter to drive EGFP permits early larval separation due to the higher expression of EGFP in the midgut in male L1 larvae compared to females [22]. Importantly, the mechanical sorting by COPAS does not significantly affect the viability and competitiveness of sorted males [21, 23]. Transgenic strains with insertions of the marker on the Y chromosome can replace the use of sex-specific promoters. Including a recombination sequence such as *attP*, *lox*, or *FRT* for site-specific integration, allows additional modification of the Y chromosome. Such strains are available for *An. gambiae* [59] and *Ae. aegypti* (Haecker et al, unpublished data).

A different strategy for sexing is pursued by the so-called X shredder. It makes use of an endonuclease, I-PpoI, that specifically cuts X-chromosomal ribosomal DNA sequences. Tissue-specific expression of I-PpoI in the testes of *An. gambiae* under the control of *β2-tub* 5’ and 3’ regulatory sequences results in a strong bias for Y gametes, as X-chromosomes are shredded during gametogenesis. However, it also resulted in male sterility in crosses of heterozygous males with wild-type females due to the transfer of the stable I-PpoI protein into gametes where it shreds the X chromosome of the oocyte upon fertilization [60]. Reducing the half-life time of the I-PpoI protein abolished the sterility effect [61].

## Site-specific modification of the *Ae. aegypti* genome

Site-specific modification of the genome can circumvent the drawbacks of random genomic integration by targeting genomic regions that have previously been shown to have no adverse effects on transgenic strains with regard to transgene function and biological quality. Two main strategies are currently used to site-specifically modify the mosquito genome, site-specific recombination systems and genome editing.

### Site-specific recombination and recombinase-mediated cassette exchange (RMCE)

Three different naturally occurring site-specific recombination systems have been turned into molecular tools to modify genomes in mammals and insects: the Cre/*lox* system from the *Escherichia coli* phage P1 [62, 63], the Flp/*FRT* system from the two-micron plasmid of *Saccharomyces cerevisiae* [64] as well as the phiC31/*att* system derived from the *Streptomyces* phage phiC31 [65]. They are all based on a recombinase enzyme that induces double-strand breaks at specific recombination sequences, followed by strand exchange and re-ligation [66]. Cre and Flp belong to the family of Serine recombinases, which recognize rather short recombination sequences, *lox* and *FRT*, respectively. These sequences have a strictly palindromic architecture with a central 8 bp core, where the strand cleavage and re-ligation occurs [67, 68]. For site-specific recombination experiments, identical recombination sequences are placed at a so-called genomic acceptor site and in a donor plasmid. Recombination leads to the integration of the donor plasmid into the genome. Due to their inverted repeat symmetry, the *lox* and *FRT* recombination sequences are preserved and can be reused for future recombination reactions. In contrast, the donor and acceptor sequences recognized by the Tyrosine recombinase phiC31 are longer and have a very limited repeat symmetry. Moreover, the donor (*attB*) and acceptor (*attP*) sequences share very little sequence identity [68, 69]. Therefore, the recombination between donor and acceptor site leads to new and incompatible *attR* and *attL* sites. *attR* and *attL* cannot recombine anymore without the presence of additional factors, thus making the reaction irreversible.

Recombination between a single donor and acceptor site leads to the integration of the complete donor plasmid at the genomic target site [70]. The concomitant integration of bacterial resistance genes and regulatory elements in the best case adds unnecessary sequence information. In the worst case it may interfere with transgene expression. To avoid this, a more sophisticated targeting strategy, the recombinase-mediated cassette exchange (RMCE), was developed. The idea for RMCE originates from the discovery that the mutation of certain bases in the central crossover region of *lox* or *FRT* sites does not abolish cleavage by the recombinase [71–73]. Instead, these mutant sites recombine with an identical (homospecific) mutant site with equal efficiency as two wild-type sites, while interaction between sites with non-identical core sequences (heterospecific sites) is prevented. For RMCE, the transgene cassette at the genomic acceptor site as well as the transgene construct in the donor plasmid is flanked by identical pairs of such heterospecific sites [68, 74]. A double recombination between the recombination sequences on both sides of the transgene constructs then leads to the exchange of the genomic landing site cassette for the gene-of-interest cassette from the donor plasmid [70, 75].

RMCE is also possible with the phiC31/*att* system. However, due to the different architecture of the recombination sites, it follows a different strategy. The genomic acceptor cassette is flanked by *attP* sites in inverted orientation, and the donor cassette is flanked by inverted *attB* sites. Double recombination between *attP* and *attB* on either side of the cassettes leads to a cassette exchange, that, again, is irreversible. It has been shown that also the opposite setup, i.e. placing the *attB* sites at the genomic landing site, and the *attP* sites in the donor plasmid, is functional [76].

Site-specific integration via a genomic *attP* docking site is the most frequently used strategy to date to site-specifically target the mosquito genome. Several *attP* docking site strains exist for the major vector species *Ae. aegypti* [77–79], *Ae. albopictus* [80], *An. gambiae* [59, 81–83], and *An*. *stephensi* [49]. Most *attP* docking site strains have been created by transposon-mediated transformation, resulting in random integration with the potential negative effects on line quality. In contrast, Bernardini et al. specifically placed an *attP* docking site on the *An*. *gambiae* Y-chromosome using meganuclease-induced homologous repair [59]. Amenya et al. tested a set of docking site strains created by transposition in *An. stephensi*. These strains carry the same construct, an *attP* docking site and marker gene. They could not detect a significant effect on mosquito fitness, suggesting that *attP* docking sites in combination with a marker gene per se do not have an inherent negative effect, as long as no insertional mutagenesis effects occur.

Site specific integration via a single *FRT* site in mosquitoes to our knowledge has been reported only in plasmid-based assays [84], and via single *lox* sites only as part of a two-step RMCE [50]. In contrast, site-specific excision via *lox* sites has been shown first by Jasinskiene et al. [85], and is commonly applied in *Ae. aegypti* [50, 79].

Successful RMCE in mosquitoes has been reported for *Ae. aegypti*, using either the Cre/*lox* or the phiC31/*att* system. Cre-RMCE was achieved in a two-step process, where the first step is comprised of the integration of the complete donor plasmid via the recombination of only one of the homospecific *lox* pairs. Injecting the resulting strain with Cre recombinase in the 2^nd^ step leads to the highly efficient excision of the plasmid backbone via recombination of the second homospecific *lox* pair, and thus to a complete cassette exchange [50].

phiC31-mediated cassette exchange was accomplished with two strategies, first with a true RMCE approach according to Bateman et al. [86], with an efficiency of four to five percent in two independent experiments [79]. The second strategy only indirectly leads to a cassette exchange in two steps by combining *attP*-mediated integration with *lox* excision. In the first step the whole donor plasmid with the cassette of interest is incorporated via single *attP* recombination, followed by *loxP* excision of the vector backbone such that only the cassette of interest remains. This strategy was termed iRMCE [79]. For *Anopheles* mosquitoes*,* only phiC31-RMCE in *An. gambiae* has been reported so far [87].

### Genome editing via TALEN and CRISPR

A different concept than site-specific recombination systems is site-specific targeting via genome editing tools. Two genome editing methods that were successfully applied to different mosquito genomes are TALEN and CRISPR. TALEs are a class of transcription activator-like effector proteins from *Xanthomona* [88, 89]. For genome editing purposes, the TAL DNA binding domain is fused to a non-specific DNA cutting enzyme, the restriction endonuclease *Fok*l [90, 91], to form the TALE nuclease (TALEN). The resulting fusion protein then specifically cuts genomic DNA at the natural binding sequence of the TAL subunit. Although the number of naturally occurring TAL binding sequences is limited, the TAL domain can theoretically be engineered to bind nearly any desired DNA sequence with relative ease [92, 93]. The method has been applied to several insect genomes [94–100] with satisfying precision and efficiency. In mosquitoes, TALEN was successfully performed in *Ae. aegypti* [99] and *An. gambiae* [100]. In both cases, the targeted gene could be knocked down due to mutations (INDELs) caused by the non-homologous end joining (NHEJ) repair mechanism at the induced double strand breaks. However, the widespread use of TALEN has been and will likely be prevented for two reasons. First, despite its relative ease, engineering TAL binding specificity for a large number of target sites still is quite work intense and costly. Second, a novel and very efficient genome editing tool based on the so-called clustered regularly interspaced short palindromic repeats (CRISPR) was developed. Using CRISPR, target site specificity is determined by the sequence of a small RNA molecule that is much easier to modify and at low cost.

CRISPR/Cas was discovered in 2007 as the prokaryotic equivalent of the eukaryotic adaptive immune system [101]. As part of their immune response to an infection, bacteria and archaea acquire short DNA sequences that originate from foreign DNA, e.g. the infecting virus or plasmid. These acquired foreign DNA sequences are incorporated into the bacterial or archaeal genome and separated by short repeat sequences, thereby forming the CRISPR. Such repeat-spacer sequences are found to date in approximately 40% and 90% of the sequenced bacterial and archaea genomes, respectively. CRISPR arrays are transcribed and processed into short CRISPR RNAs (crRNA) by nucleases of the Cas (CRISPR associated) family of proteins. Small clusters of Cas genes are located next to the CRISPR arrays. The crRNAs are incorporated into ribonucleoprotein (RNP) complexes with another class of Cas proteins. The CRISPR RNPs recognize foreign DNA by sequence complementarity with the crRNA. The foreign DNA is then cut by a Cas nuclease activity similar to RNA interference in eukaryotic organisms [102], thus conferring immunity against infection with previously encountered infective agents (see [102, 103] or [104] for comprehensive explanations and illustrations).

Of the three different CRISPR/Cas systems known so far, the multifunctional Cas9 protein from the type II system is the most used nuclease for genome editing in insects. Cas9 requires two types of RNA to function, the crRNA and a transactivating CRISPR RNA (tracrRNA). For more convenient use, scientists engineered the crRNA and tracrRNA into one single-guide RNA (sgRNA) [105]. Moreover, Cas9 activity depends on the presence of a so-called protospacer adjacent motif (PAM) next to the target site. Since its discovery, the CRISPR genome editing system has seen a rapid development and constant improvement of efficiency and specificity by engineering the existing Cas protein [106–110], characterizing and adapting new Cas proteins like Cpf1 [111] or C2c2 [112], or by engineering of sgRNAs [113, 114]. For a comprehensive review on current Cas variants see Cebrian-Serrano et al. [115].

The first insect genome edited via CRISPR/Cas was that of *D. melanogaster* [116–118]. In 2014 and 2015, CRISPR/Cas was transferred to mosquitoes. Dong et al. provided the first proof-of-principle experiment in *Ae. aegypti* by knocking out a marker gene in a transgenic line [119]. At the same time, Kistler et al. published a systematic study on the composition of CRISPR injection mixes and gRNA design in *Ae. aegypti*. The authors achieved a knockdown rate of at least 18% using two gRNAs per target gene in three independent experiments. Moreover, they succeeded in integrating large fluorescent protein cassettes via CRISPR/Cas [120]. Recently it was shown that germline expression of Cas9 in *Ae. aegypti* strongly enhances the mutagenesis rate [121], in accordance with the results in *D. melanogaster* [122–125]. In 2015, Basu et al. investigated the effect of knocking down the NHEJ pathway in *Ae. aegypti* on the rate of NHEJ versus HDR in TALEN and CRISPR experiments [126]. The permanent knockdown of *Lig4* failed to create strains viable for more than a few generations and transient *Lig4* knockdown did not produce the desired results. In contrast, the transient knockdown of *ku70* (which was also successfully used in *B. mori* to suppress NHEJ [127]) resulted in HDR events with a frequency of about 2% for TALEN as well as CRISPR [126].

With the simplicity to re-program CRISPR/Cas to new target sites, it has become much easier and more straight-forward to knock down specific genes to elucidate their function. During the investigation of the gene *Nix* as the long-searched *Aedes* maleness-factor, CRISPR/Cas was used to knock down *Nix* to show that the factor governs the male determining pathway. Its knockdown resulted in female-specific splicing of the sex-determination genes *fruitless* and *doublesex*, and feminized genetic males [20]. Similarly, CRISPR knockdown of two miRNA-encoding sequences in *Ae*. *aegypti* helped to elucidate their function in development and lipid metabolism [128, 129].

Along the same line, Li et al. optimized CRISPR for site-specific mutagenesis in three *Anopheles* species, *An. funestus, An. coluzii* and *An*. *albimanus,* with the goal to establish CRISPR/Cas as an efficient tool for reverse genetics in *Anopheles* mosquitoes [130]. In *An. gambiae,* CRISPR/Cas was used to target ribosomal sequences on the X chromosome, with the goal of creating a sexing strain. Expressing Cas9 during male gametogenesis leads to RNA-guided shredding of the X chromosome in sperm, resulting in highly male-biased offspring [131]. This experiment recapitulates the original X-shredder that used a naturally occurring endonuclease targeting ribosomal sequences on the X chromosome [61]. Recently, a pipeline for the identification of abundant and specific X-chromosome target sequences for X-shredding by CRISPR endonucleases was developed [132]. The flexibility of CRISPR/Cas should allow the transfer of this approach to many other mosquito species with an XY sex determination system.

Besides strongly facilitating functional gene studies and genome editing, CRISPR has also revolutionized the field of gene drive research. Gene drives offer additional possibilities to address population control or vector capacity of mosquitoes. They could be developed to drive female sterility into a population, or to convert females into males, both of which should lead to the collapse of the (local) wild population. On the other hand, natural populations could be replaced with transgenic strains that are refractive to pathogen infection [48, 133–135]. Recent work resulted in two gene drive systems in *Anopheles*. The first approach in *An. gambiae* targets female fertility by inserting a CRISPR/Cas gene drive construct in three candidate genes conferring recessive female sterility upon disruption [87]. A CRISPR-based gene drive in *An. stephensi* is used to drive multiple anti-plasmodium effector genes into a wildtype population, resulting in more than 99% of positive offspring [136]. Gene drive research, however, is still at its beginning. Consequently, the understanding of gene drive behavior in a real population is very limited, especially since each drive behaves differently, depending on its architecture and components. Although there is lots of activity on the modeling side to predict the behavior of gene drives in a population [137–143], there are not many real data sets available yet. Moreover, recently observed resistance development poses further challenges for the field [144–146]. Therefore, more thorough research including safeguards, control strategies and potential reversibility of released gene drives will be necessary before such applications could be considered for vector control [147].

## Conclusions

With transposable elements, site-specific recombination systems and genome editing via CRISPR/Cas there are three powerful molecular tools available that can help to achieve the goal of creating efficient sexing strains for the major vector mosquitoes and thereby enable large-scale control programs based on the (mass-) release of male mosquitoes. *Aedes* as well as *Anopheles* mosquitoes are amenable to the available tools, and researchers are continuously working to further develop and optimize the tools for their use in efficient mosquito genome modification [83, 120, 121, 148]. These molecular tools can be used in two ways to progress towards the goal of creating sexing strains in mosquitoes. First, in basic research and second, in the application of the knowledge obtained from basic research to build sexing systems. Basic research recently led to the discovery of important male-determining factors in mosquitoes, the *Ae. aegypti* M-factor *Nix* [20], and the *An*. *gambiae* maleness factor *Yob* [149]. These crucial insights into mosquito sex determination can and will be exploited for the development of sexing systems, for example to build a female lethality system by conditionally overexpressing *Yob* [149], or to achieve female to male conversion by conditionally overexpressing *Nix* [20]. The available genome modification tools will then allow the creation of (transgenic) sexing strains for vector control applications. Other strategies could be to a build a female lethality system similar to the TESS, based on the conditional early embryonic expression of lethal genes in females but not males [24, 25]. While it has been shown that exogenous genes and regulatory elements for such constructs can be functional in related species [24, 25, 54], several studies indicated that the endogenous homologue, if available, can be more effective [55]. Where such homologues have not been identified yet or for species where no genome sequencing data are available, more basic research will be necessary. Alternatively, mutations that are already successfully used for sexing in one species, could be exploited to create similar mutations in other species via CRISPR, once the molecular basis of the mutation is uncovered. The resulting sexing strains might even not be considered transgenic, depending on the applied modification. A prime example for such a mutation is the Medfly VIENNA 8 *temperature*-*sensitive lethal* (*tsl*) that was created by chemical mutagenesis and kills female embryos upon heat shock [150]. Genome modification and genome editing tools could thus facilitate the transfer of successful strategies from one species to another. Site-specific systems further allow to compare the effectiveness of potential candidate genes and transgene constructs for sexing systems at the same genomic location unbiased by genomic position effects, which is not possible for transgenic strains created by transposition due to the random genomic integration. Thus, the most suitable approach or construct in terms of sexing efficacy can be selected.

## Abbreviations

*β2-tub*: *beta2-tubulin*
Cas: CRISPR associated protein
COPAS: Complex Object Parametric Analyzer and Sorter
CRISPR: clustered regularly interspaced short palindromic repeats
crRNA: CRISPR RNA
*Dsx*: *doublesex*
EGFP: enhanced green fluorescent protein
Flp: Flipase
GSS: genetic sexing strain
HDR: homology-directed repair
*Hid*: *head involution defective*
INDEL: insertion/deletion
ITR: inverted terminal repeat
NHEJ: non-homologous end joining
PAM: protospacer adjacent motif
PCR: polymerase chain reaction
RMCE: recombinase-mediated cassette exchange
Ser: serine
sgRNA: single guide RNA
SIT: sterile insect technique
TALE: transcription activator-like effector
TALEN: transcription activator-like effector nuclease
TESS: transgenic embryonic sexing strain
tracrRNA: transactivating CRISPR RNA
*tsl*: *temperature sensitive lethal*
TSS: transgenic sexing strain
Tyr: tyrosine

## References

[1] Vreysen MJ, Saleh KM, Ali MY, Abdulla AM, Zhu ZR, Juma KG (2000). *Glossina austeni* (Diptera: Glossinidae) eradicated on the island of Unguja, Zanzibar, using the sterile insect technique. J Econ Entomol.

[2] Wyss JH (2000). Screwworm eradication in the Americas - overview. Edited by Tan K.

[3] Hendrichs J, Ortiz G, Liedo P, Schwarz A, Cavalloro R (1983). Six years of successful medfly program in Mexico and Guatemala. Proceedings, Symposium: fruit flies of economic importance CEC/IOBC International Symposium, 16-19 November 1982, Athens, Greece.

[4] Villasenor A, Carrillo J, Zavala J, Stewart J, Lira C, Reyes J, Hong TK (2000). Current progress in the medfly program Mexico-Guatemala. Area-wide management of fruit flies and other major insect pests.

[5] Dowell RV, Siddiqui IA, Meyer F, Spaugy EL (1999). Early results suggest sterile flies may protect S. California from Medfly. California Agriculture.

[6] Dowell RV, Siddiqui IA, Meyer F, Spaugy EL, Tan KH (2000). Mediterranean fruit fly preventive release program in southern California. Area-wide control of fruit flies and other insect pests.

[7] CDFA (2000). Mediterranean fruit fly preventive release program. Report to the Legislature. Plant health & pest prevention services of the California department of food and agriculture.

[8] Rendon P, McInnis D, Lance D, Stewart J (2004). Medfly (Diptera: Tephritidae) genetic sexing: large-scale field comparison of males-only and bisexual sterile fly releases in Guatemala. J Econ Entomol.

[9] Harris AF, McKemey AR, Nimmo D, Curtis Z, Black I, Morgan SA (2012). Successful suppression of a field mosquito population by sustained release of engineered male mosquitoes. Nat Biotechnol.

[10] Harris AF, Nimmo D, McKemey AR, Kelly N, Scaife S, Donnelly CA, Beech C, Petrie WD, Alphey L (2011). Field performance of engineered male mosquitoes. Nat Biotechnol.

[11] Carvalho DO, McKemey AR, Garziera L, Lacroix R, Donnelly CA, Alphey L, Malavasi A, Capurro ML (2015). Suppression of a field population of *Aedes aegypti* in Brazil by sustained release of transgenic male mosquitoes. PLoS Negl Trop Dis.

[12] Garziera L, Pedrosa MC, Almeida de Souza F, Gómez M, Moreira MB, Virginio JF, Capurro ML, Carvalho DO (2017). Effect of interruption of over-flooding releases of transgenic mosquitoes over wild population of *Aedes aegypti*: two case studies in Brazil. Entomologia Experimentalis et Applicata.

[13] Phuc HK, Andreasen MH, Burton RS, Vass C, Epton MJ, Pape G (2007). Late-acting dominant lethal genetic systems and mosquito control. BMC Biol.

[14] Carvalho DO, Nimmo D, Naish N, McKemey AR, Gray P, Wilke AB (2014). Mass production of genetically modified *Aedes aegypti* for field releases in Brazil. J Vis Exp.

[15] Papathanos PA, Bossin HC, Benedict MQ, Catteruccia F, Malcolm CA, Alphey L, Crisanti A (2009). Sex separation strategies: past experience and new approaches. Malar J.

[16] Gilles JR, Schetelig MF, Scolari F, Marec F, Capurro ML, Franz G, Bourtzis K (2014). Towards mosquito sterile insect technique programmes: exploring genetic, molecular, mechanical and behavioural methods of sex separation in mosquitoes. Acta Trop.

[17] Yamada H, Benedict MQ, Malcolm CA, Oliva CF, Soliban SM, Gilles JR (2012). Genetic sex separation of the malaria vector, *Anopheles arabiensis*, by exposing eggs to dieldrin. Malar J.

[18] Curtis CF, Akiyama J, Davidson G (1976). A genetic sexing system in *Anopheles gambiae* species A. Mosquito News.

[19] Kaiser PE, Seawright JA, Dame DA, Joslyn DJ (1978). Development of a genetic sexing system for *Anopheles albimanus*. J Econ Entomol.

[20] Hall AB, Basu S, Jiang X, Qi Y, Timoshevskiy VA, Biedler JK (2015). Sex determination. A male-determining factor in the mosquito *Aedes aegypti*. Science.

[21] Catteruccia F, Benton JP, Crisanti A (2005). An *Anopheles* transgenic sexing strain for vector control. Nat Biotechnol.

[22] Marois E, Scali C, Soichot J, Kappler C, Levashina EA, Catteruccia F (2012). High-throughput sorting of mosquito larvae for laboratory studies and for future vector control interventions. Malar J.

[23] Smith RC, Walter MF, Hice RH, O'Brochta DA, Atkinson PW (2007). Testis-specific expression of the *β2 tubulin* promoter of *Aedes aegypti* and its application as a genetic sex-separation marker. Insect Molecular Biology.

[24] Ogaugwu CE, Schetelig MF, Wimmer EA (2013). Transgenic sexing system for *Ceratitis capitata* (Diptera: Tephritidae) based on female-specific embryonic lethality. Insect Biochem Mol Biol.

[25] Schetelig MF, Handler AM (2012). A transgenic embryonic sexing system for *Anastrepha suspensa* (Diptera: Tephritidae). Insect Biochem Mol Biol.

[26] Yan Y, Scott MJ (2015). A transgenic embryonic sexing system for the Australian sheep blow fly *Lucilia cuprina*. Sci Rep.

[27] Rubin GM, Spradling AC (1982). Genetic transformation of *Drosophila* with transposable element vectors. Science.

[28] Kokoza V, Ahmed A, Wimmer EA, Raikhel AS (2001). Efficient transformation of the yellow fever mosquito *Aedes aegypti* using the *piggyBac* transposable element vector pBac[3xP3-EGFPafm]. Insect Biochem Mol Biol.

[29] Palavesam A, Esnault C, O'Brochta DA (2013). Post-integration silencing of *piggyBac* transposable elements in *Aedes aegypti*. PLoS One.

[30] Sethuraman N, Fraser MJ, Eggleston P, O'Brochta DA (2007). Post-integration stability of *piggyBac* in *Aedes aegypti*. Insect Biochemistry and Molecular Biology.

[31] Esnault C, Palavesam A, Pilitt K, O'Brochta DA (2011). Intrinsic characteristics of neighboring DNA modulate transposable element activity in *Drosophila melanogaster*. Genetics.

[32] Lobo N, Li X, Hua-Van A, Fraser MJ (2001). Mobility of the *piggyBac* transposon in embryos of the vectors of Dengue fever (*Aedes albopictus*) and La Crosse encephalitis (*Ae. triseriatus*). Mol Genet Genomics.

[33] O'Brochta DA, Sethuraman N, Wilson R, Hice RH, Pinkerton AC, Levesque CS (2003). Gene vector and transposable element behavior in mosquitoes. J Exp Biol.

[34] Smith RC, Atkinson PW (2011). Mobility properties of the *Hermes* transposable element in transgenic lines of *Aedes aegypti*. Genetica.

[35] Wilson R, Orsetti J, Klocko AD, Aluvihare C, Peckham E, Atkinson PW, Lehane MJ, O'Brochta DA (2003). Post-integration behavior of a *Mos1* mariner gene vector in *Aedes aegypti*. Insect Biochem Mol Biol.

[36] Guimond N, Bideshi DK, Pinkerton AC, Atkinson PW, O'Brochta DA (2003). Patterns of *Hermes* transposition in *Drosophila melanogaster*. Mol Genet Genomics.

[37] Lidholm DA, Lohe AR, Hartl DL (1993). The transposable element *mariner* mediates germline transformation in *Drosophila melanogaster*. Genetics.

[38] Scali C, Nolan T, Sharakhov I, Sharakhova M, Crisanti A, Catteruccia F (2007). Post-integration behavior of a *Minos* transposon in the malaria mosquito *Anopheles stephensi*. Mol Genet Genomics.

[39] O'Brochta DA, Alford RT, Pilitt KL, Aluvihare CU, Harrell RA (2011). 2nd. *piggyBac* transposon remobilization and enhancer detection in *Anopheles* mosquitoes. Proc Natl Acad Sci USA.

[40] O'Brochta DA, Pilitt KL, Harrell RA, Aluvihare C, Alford RT (2012). Gal4-based enhancer-trapping in the malaria mosquito *Anopheles stephensi*. G3 (Bethesda).

[41] Cadinanos J, Bradley A (2007). Generation of an inducible and optimized *piggyBac* transposon system. Nucleic Acids Res.

[42] Liang Q, Kong J, Stalker J, Bradley A (2009). Chromosomal mobilization and reintegration of *Sleeping Beauty* and *PiggyBac* transposons. Genesis.

[43] Yusa K, Zhou L, Li MA, Bradley A, Craig NL (2011). A hyperactive *piggyBac* transposase for mammalian applications. Proc Natl Acad Sci USA.

[44] Wright JA, Smith RC, Li X, Craig NL, Atkinson PW (2013). IPB7 transposase behavior in *Drosophila melanogaster* and *Aedes aegypti*. Insect Biochem Mol Biol.

[45] Eckermann KN, Ahmed HMM, KaramiNejadRanjbar M, Dippel S, Ogaugwu CE, Kitzmann P, Isah MD, Wimmer EA (2018). Hyperactive piggyBac transposase improves transformation efficiency in diverse insect species. Insect Biochem Mol Biol.

[46] Lampe DJ, Akerley BJ, Rubin EJ, Mekalanos JJ, Robertson HM (1999). Hyperactive transposase mutants of the *Himar1* mariner transposon. Proc Natl Acad Sci U S A.

[47] Pledger DW, Coates CJ (2005). Mutant *Mos1* mariner transposons are hyperactive in *Aedes aegypti*. Insect Biochem Mol Biol.

[48] Moreira LA, Wang J, Collins FH, Jacobs-Lorena M (2004). Fitness of anopheline mosquitoes expressing transgenes that inhibit *Plasmodium* development. Genetics.

[49] Amenya DA, Bonizzoni M, Isaacs AT, Jasinskiene N, Chen H, Marinotti O, Yan G, James AA (2010). Comparative fitness assessment of *Anopheles stephensi* transgenic lines receptive to site-specific integration. Insect Mol Biol.

[50] Häcker I, Harrell RA, Eichner G, Pilitt KL, O'Brochta DA, Handler AM, Schetelig MF (2017). Cre/lox-recombinase-mediated cassette exchange for reversible site-specific genomic targeting of the disease vector *Aedes aegypti*. Sci Rep.

[51] Catteruccia F, Godfray HC, Crisanti A (2003). Impact of genetic manipulation on the fitness of *Anopheles stephensi* mosquitoes. Science.

[52] Irvin N, Hoddle MS, O'Brochta DA, Carey B, Atkinson PW (2004). Assessing fitness costs for transgenic *Aedes aegypti* expressing the GFP marker and transposase genes. Proc Natl Acad Sci U S A.

[53] Ito J, Ghosh A, Moreira LA, Wimmer EA, Jacobs-Lorena M (2002). Transgenic anopheline mosquitoes impaired in transmission of a malaria parasite. Nature.

[54] Schetelig MF, Caceres C, Zacharopoulou A, Franz G, Wimmer EA (2009). Conditional embryonic lethality to improve the sterile insect technique in *Ceratitis capitata* (Diptera: Tephritidae). BMC Biol.

[55] Schetelig MF, Handler AM (2012). Strategy for enhanced transgenic strain development for embryonic conditional lethality in *Anastrepha suspensa*. Proc Natl Acad Sci USA.

[56] Schetelig MF, Targovska A, Meza JS, Bourtzis K, Handler AM. Tetracycline-suppressible female lethality and sterility in the Mexican fruit fly, *Anastrepha ludens*. Insect Mol Biol. 2016;25(4):500–8. 10.1111/imb.12238 27135433

[57] Robertson G, Garrick D, Wu W, Kearns M, Martin D, Whitelaw E (1995). Position-dependent variegation of globin transgene expression in mice. Proc Natl Acad Sci U S A.

[58] Elgin SC, Reuter G (2013). Position-effect variegation, heterochromatin formation, and gene silencing in *Drosophila*. Cold Spring Harb Perspect Biol.

[59] Bernardini F, Galizi R, Menichelli M, Papathanos PA, Dritsou V, Marois E, Crisanti A, Windbichler N (2014). Site-specific genetic engineering of the *Anopheles gambiae* Y chromosome. Proc Natl Acad Sci USA.

[60] Windbichler N, Papathanos PA, Crisanti A (2008). Targeting the X chromosome during spermatogenesis induces Y chromosome transmission ratio distortion and early dominant embryo lethality in *Anopheles gambiae*. PLoS Genet.

[61] Galizi R, Doyle LA, Menichelli M, Bernardini F, Deredec A, Burt A, Stoddard BL, Windbichler N, Crisanti A (2014). A synthetic sex ratio distortion system for the control of the human malaria mosquito. Nat Commun.

[62] Siegal ML, Hartl DL (2000). Application of Cre/*loxP* in *Drosophila*. Site-specific recombination and transgene coplacement. Methods in Molecular Biology.

[63] Siegal ML, Hartl DL (1996). Transgene coplacement and high efficiency site-specific recombination with the Cre/*loxP* system in *Drosophila*. Genetics.

[64] Andrews BJ, Beatty LG, Sadowski PD (1986). Site-specific recombination of the yeast plasmid two-micron circle: intermediates in the binding process. Basic Life Sciences.

[65] Thorpe HM, Smith MC (1998). *In vitro* site-specific integration of bacteriophage DNA catalyzed by a recombinase of the resolvase/invertase family. Proceedings of the National Academy of Sciences, USA.

[66] Grindley ND, Whiteson KL, Rice PA (2006). Mechanisms of site-specific recombination. Annu Rev Biochem.

[67] Kilby NJ, Snaith MR, Murray JA (1993). Site-specific recombinases: tools for genome engineering. Trends Genet.

[68] Turan S, Bode J (2011). Site-specific recombinases: from tag-and-target- to tag-and-exchange-based genomic modifications. FASEB J.

[69] Rausch H, Lehmann M (1991). Structural analysis of the actinophage phi C31 attachment site. Nucleic Acids Res.

[70] Turan S, Zehe C, Kuehle J, Qiao J, Bode J (2013). Recombinase-mediated cassette exchange (RMCE) - a rapidly-expanding toolbox for targeted genomic modifications. Gene.

[71] Hoess RH, Wierzbicki A, Abremski K (1986). The role of the *loxP* spacer region in P1 site-specific recombination. Nucleic Acids Res.

[72] Lee G, Saito I (1998). Role of nucleotide sequences of *loxP* spacer region in Cre-mediated recombination. Gene.

[73] Missirlis PI, Smailus DE, Holt RA (2006). A high-throughput screen identifying sequence and promiscuity characteristics of the *loxP* spacer region in Cre-mediated recombination. BMC Genomics.

[74] Schlake T, Bode J (1994). Use of mutated FLP recognition target (*FRT*) sites for the exchange of expression cassettes at defined chromosomal loci. Biochemistry.

[75] Turan S, Galla M, Ernst E, Qiao J, Voelkel C, Schiedlmeier B, Zehe C, Bode J (2011). Recombinase-mediated cassette exchange (RMCE): traditional concepts and current challenges. J Mol Biol.

[76] Long D, Zhao A, Xu L, Lu W, Guo Q, Zhang Y, Xiang Z (2013). In vivo site-specific integration of transgene in silkworm via PhiC31 integrase-mediated cassette exchange. Insect Biochem Mol Biol.

[77] Nimmo DD, Alphey L, Meredith JM, Eggleston P (2006). High efficiency site-specific genetic engineering of the mosquito genome. Insect Molecular Biology.

[78] Franz AW, Jasinskiene N, Sanchez-Vargas I, Isaacs AT, Smith MR, Khoo CC, Heersink MS, James AA, Olson KE (2011). Comparison of transgene expression in *Aedes aegypti* generated by mariner *Mos1* transposition and PhiC31 site-directed recombination. Insect Mol Biol.

[79] Haghighat-Khah RE, Scaife S, Martins S, St John O, Matzen KJ, Morrison N, Alphey L (2015). Site-specific cassette exchange systems in the *Aedes aegypti* mosquito and the *Plutella xylostella* moth. PLoS One.

[80] Labbé GM, Nimmo DD, Alphey L (2010). *piggybac*- and PhiC31-mediated genetic transformation of the Asian tiger mosquito, *Aedes albopictus* (Skuse). PLoS neglected tropical diseases.

[81] Meredith JM, Basu S, Nimmo DD, Larget-Thiery I, Warr EL, Underhill A (2011). Site-specific integration and expression of an anti-malarial gene in transgenic *Anopheles gambiae* significantly reduces *Plasmodium* infections. PLoS One.

[82] Meredith JM, Underhill A, McArthur CC, Eggleston P (2013). Next-generation site-directed transgenesis in the malaria vector mosquito *Anopheles gambiae*: self-docking strains expressing germline-specific phiC31 integrase. PLoS One.

[83] Volohonsky G, Terenzi O, Soichot J, Naujoks DA, Nolan T, Windbichler N (2015). Tools for *Anopheles gambiae* transgenesis. G3 (Bethesda).

[84] Morris AC, Schaub TL, James AA (1991). FLP-mediated recombination in the vector mosquito *Aedes aegypti*. Nucleic Acids Res.

[85] Jasinskiene N, Coates CJ, Ashikyan A, James AA (2003). High efficiency, site-specific excision of a marker gene by the phage P1 cre-loxP system in the yellow fever mosquito *Aedes aegypti*. Nucleic Acids Res.

[86] Bateman JR, Lee AM, Wu CT (2006). Site-specific transformation of Drosophila via phiC31 integrase-mediated cassette exchange. Genetics.

[87] Hammond A, Galizi R, Kyrou K, Simoni A, Siniscalchi C, Katsanos D (2016). A CRISPR-Cas9 gene drive system targeting female reproduction in the malaria mosquito vector *Anopheles gambiae*. Nat Biotechnol.

[88] Boch J, Scholze H, Schornack S, Landgraf A, Hahn S, Kay S, Lahaye T, Nickstadt A, Bonas U (2009). Breaking the code of DNA binding specificity of TAL-type III effectors. Science.

[89] Moscou MJ, Bogdanove AJ (2009). A simple cipher governs DNA recognition by TAL effectors. Science.

[90] Kim YG, Cha J, Chandrasegaran S (1996). Hybrid restriction enzymes: zinc finger fusions to Fok I cleavage domain. Proc Natl Acad Sci USA.

[91] Li T, Huang S, Jiang WZ, Wright D, Spalding MH, Weeks DP, Yang B (2011). TAL nucleases (TALNs): hybrid proteins composed of TAL effectors and FokI DNA-cleavage domain. Nucleic Acids Res.

[92] Geissler R, Scholze H, Hahn S, Streubel J, Bonas U, Behrens SE, Boch J (2011). Transcriptional activators of human genes with programmable DNA-specificity. PLoS One.

[93] Reyon D, Tsai SQ, Khayter C, Foden JA, Sander JD, Joung JK (2012). FLASH assembly of TALENs for high-throughput genome editing. Nat Biotechnol.

[94] Rong YS, Golic KG (2001). A targeted gene knockout in *Drosophila*. Genetics.

[95] Bozas A, Beumer KJ, Trautman JK, Carroll D (2009). Genetic analysis of zinc-finger nuclease-induced gene targeting in *Drosophila*. Genetics.

[96] Takasu Y, Kobayashi I, Beumer K, Uchino K, Sezutsu H, Sajwan S, Carroll D, Tamura T, Zurovec M (2010). Targeted mutagenesis in the silkworm *Bombyx mori* using zinc finger nuclease mRNA injection. Insect Biochem Mol Biol.

[97] Takasu Y, Sajwan S, Daimon T, Osanai-Futahashi M, Uchino K, Sezutsu H, Tamura T, Zurovec M (2013). Efficient TALEN construction for *Bombyx mori* gene targeting. PLoS One.

[98] Liu J, Li C, Yu Z, Huang P, Wu H, Wei C (2012). Efficient and specific modifications of the *Drosophila* genome by means of an easy TALEN strategy. J Genet Genomics.

[99] Aryan A, Anderson MA, Myles KM, Adelman ZN (2013). TALEN-based gene disruption in the dengue vector *Aedes aegypti*. PLoS One.

[100] Smidler AL, Terenzi O, Soichot J, Levashina EA, Marois E (2013). Targeted mutagenesis in the malaria mosquito using TALE nucleases. PLoS One.

[101] Barrangou R, Fremaux C, Deveau H, Richards M, Boyaval P, Moineau S, Romero DA, Horvath P (2007). CRISPR provides acquired resistance against viruses in prokaryotes. Science.

[102] Marraffini LA, Sontheimer EJ (2010). CRISPR interference: RNA-directed adaptive immunity in bacteria and archaea. Nat Rev Genet.

[103] Horvath P, Barrangou R (2010). CRISPR/Cas, the immune system of bacteria and archaea. Science.

[104] Bhaya D, Davison M, Barrangou R (2011). CRISPR-Cas systems in bacteria and archaea: versatile small RNAs for adaptive defense and regulation. Annu Rev Genet.

[105] Jinek M, Chylinski K, Fonfara I, Hauer M, Doudna JA, Charpentier E (2012). A programmable dual-RNA-guided DNA endonuclease in adaptive bacterial immunity. Science.

[106] Davis L, Maizels N (2014). Homology-directed repair of DNA nicks via pathways distinct from canonical double-strand break repair. Proc Natl Acad Sci USA.

[107] Ran FA, Hsu PD, Lin CY, Gootenberg JS, Konermann S, Trevino AE (2013). Double nicking by RNA-guided CRISPR Cas9 for enhanced genome editing specificity. Cell.

[108] Cong L, Ran FA, Cox D, Lin S, Barretto R, Habib N (2013). Multiplex genome engineering using CRISPR/Cas systems. Science.

[109] Qi LS, Larson MH, Gilbert LA, Doudna JA, Weissman JS, Arkin AP, Lim WA (2013). Repurposing CRISPR as an RNA-guided platform for sequence-specific control of gene expression. Cell.

[110] Kleinstiver BP, Pattanayak V, Prew MS, Tsai SQ, Nguyen NT, Zheng Z, Joung JK (2016). High-fidelity CRISPR-Cas9 nucleases with no detectable genome-wide off-target effects. Nature.

[111] Zetsche B, Gootenberg JS, Abudayyeh OO, Slaymaker IM, Makarova KS, Essletzbichler P (2015). Cpf1 is a single RNA-guided endonuclease of a class 2 CRISPR-Cas system. Cell.

[112] Abudayyeh OO, Gootenberg JS, Konermann S, Joung J, Slaymaker IM, Cox DB (2016). C2c2 is a single-component programmable RNA-guided RNA-targeting CRISPR effector. Science.

[113] Fu Y, Sander JD, Reyon D, Cascio VM, Joung JK (2014). Improving CRISPR-Cas nuclease specificity using truncated guide RNAs. Nat Biotechnol.

[114] Nowak CM, Lawson S, Zerez M, Bleris L. Guide RNA engineering for versatile Cas9 functionality. Nucleic Acids Res. 2016;44(20):9555–64. 10.1093/nar/gkw908 PMC5175371 27733506

[115] Cebrian-Serrano A, Davies B (2017). CRISPR-Cas orthologues and variants: optimizing the repertoire, specificity and delivery of genome engineering tools. Mamm Genome.

[116] Ren X, Sun J, Housden BE, Hu Y, Roesel C, Lin S (2013). Optimized gene editing technology for *Drosophila melanogaster* using germ line-specific Cas9. Proc Natl Acad Sci U S A.

[117] Yu Z, Ren M, Wang Z, Zhang B, Rong YS, Jiao R, Gao G (2013). Highly efficient genome modifications mediated by CRISPR/Cas9 in *Drosophila*. Genetics.

[118] Bassett AR, Tibbit C, Ponting CP, Liu JL (2013). Highly efficient targeted mutagenesis of *Drosophila* with the CRISPR/Cas9 system. Cell Rep.

[119] Dong S, Lin J, Held NL, Clem RJ, Passarelli AL, Franz AW (2015). Heritable CRISPR/Cas9-mediated genome editing in the yellow fever mosquito *Aedes aegypti*. PLoS One.

[120] Kistler KE, Vosshall LB, Matthews BJ (2015). Genome engineering with CRISPR-Cas9 in the mosquito *Aedes aegypti*. Cell Rep.

[121] Li M, Bui M, Yang T, Bowman CS, White BJ, Akbari OS (2017). Germline Cas9 expression yields highly efficient genome engineering in a major worldwide disease vector, *Aedes aegypti*. Proc Natl Acad Sci USA.

[122] Sebo ZL, Lee HB, Peng Y, Guo Y (2014). A simplified and efficient germline-specific CRISPR/Cas9 system for *Drosophila* genomic engineering. Fly (Austin).

[123] Port F, Chen HM, Lee T, Bullock SL (2014). Optimized CRISPR/Cas tools for efficient germline and somatic genome engineering in *Drosophila*. Proc Natl Acad Sci U S A.

[124] Kondo S, Ueda R (2013). Highly improved gene targeting by germline-specific Cas9 expression in *Drosophila*. Genetics.

[125] Gratz SJ, Ukken FP, Rubinstein CD, Thiede G, Donohue LK, Cummings AM, O'Connor-Giles KM (2014). Highly specific and efficient CRISPR/Cas9-catalyzed homology-directed repair in *Drosophila*. Genetics.

[126] Basu S, Aryan A, Overcash JM, Samuel GH, Anderson MA, Dahlem TJ, Myles KM, Adelman ZN (2015). Silencing of end-joining repair for efficient site-specific gene insertion after TALEN/CRISPR mutagenesis in *Aedes aegypti*. Proc Natl Acad Sci U S A.

[127] Ma S, Chang J, Wang X, Liu Y, Zhang J, Lu W (2014). CRISPR/Cas9 mediated multiplex genome editing and heritable mutagenesis of BmKu70 in *Bombyx mori*. Sci Rep.

[128] Ling L, Kokoza VA, Zhang C, Aksoy E, Raikhel AS (2017). MicroRNA-277 targets insulin-like peptides 7 and 8 to control lipid metabolism and reproduction in *Aedes aegypti* mosquitoes. Proc Natl Acad Sci USA.

[129] Zhang Y, Zhao B, Roy S, Saha TT, Kokoza VA, Li M, Raikhel AS (2016). microRNA-309 targets the Homeobox gene SIX4 and controls ovarian development in the mosquito *Aedes aegypti*. Proc Natl Acad Sci USA.

[130] Li M, Akbari OS, White BJ. Highly efficient site-specific mutagenesis in malaria mosquitoes using CRISPR. G3 (Bethesda). 2018;8(2):653–8. 10.1534/g3.117.1134 PMC5919725 29233915

[131] Galizi R, Hammond A, Kyrou K, Taxiarchi C, Bernardini F, O'Loughlin SM (2016). A CRISPR-Cas9 sex-ratio distortion system for genetic control. Sci Rep.

[132] Papathanos PA, Windbichler N (2018). An assembly-free pipeline for the identification of abundant and specific X-chromosome target sequences for X-shredding by CRISPR endonucleases. The CRISPR Journal.

[133] Volohonsky G, Hopp AK, Saenger M, Soichot J, Scholze H, Boch J, Blandin SA, Marois E (2017). Transgenic expression of the anti-parasitic factor TEP1 in the malaria mosquito *Anopheles gambiae*. PLoS Pathog.

[134] McArthur CC, Meredith JM, Eggleston P (2014). Transgenic *Anopheles gambiae* expressing an antimalarial peptide suffer no significant fitness cost. PLoS One.

[135] Sumitani M, Kasashima K, Yamamoto DS, Yagi K, Yuda M, Matsuoka H, Yoshida S (2013). Reduction of malaria transmission by transgenic mosquitoes expressing an antisporozoite antibody in their salivary glands. Insect Mol Biol.

[136] Gantz VM, Jasinskiene N, Tatarenkova O, Fazekas A, Macias VM, Bier E, James AA (2015). Highly efficient Cas9-mediated gene drive for population modification of the malaria vector mosquito *Anopheles stephensi*. Proc Natl Acad Sci USA.

[137] Tanaka H, Stone HA, Nelson DR (2017). Spatial gene drives and pushed genetic waves. Proc Natl Acad Sci USA.

[138] Deredec A, Burt A, Godfray HC (2008). The population genetics of using homing endonuclease genes in vector and pest management. Genetics.

[139] Deredec A, Godfray HC, Burt A (2011). Requirements for effective malaria control with homing endonuclease genes. Proc Natl Acad Sci USA.

[140] North A, Burt A, Godfray HC, Buckley Y (2013). Modelling the spatial spread of a homing endonuclease gene in a mosquito population. J Appl Ecol.

[141] Beaghton A, Beaghton PJ, Burt A (2016). Gene drive through a landscape: reaction-diffusion models of population suppression and elimination by a sex ratio distorter. Theor Popul Biol.

[142] Marshall JM, Hay BA (2012). Confinement of gene drive systems to local populations: a comparative analysis. J Theor Biol.

[143] Marshall JM, Hay BA (2012). General principles of single-construct chromosomal gene drive. Evolution.

[144] Hammond AM, Kyrou K, Bruttini M, North A, Galizi R, Karlsson X (2017). The creation and selection of mutations resistant to a gene drive over multiple generations in the malaria mosquito. PLoS Genet.

[145] Noble C, Olejarz J, Esvelt KM, Church GM, Nowak MA (2017). Evolutionary dynamics of CRISPR gene drives. Sci Adv.

[146] Champer J, Reeves R, Oh SY, Liu C, Liu J, Clark AG, Messer PW (2017). Novel CRISPR/Cas9 gene drive constructs reveal insights into mechanisms of resistance allele formation and drive efficiency in genetically diverse populations. PLoS Genet.

[147] Esvelt KM, Smidler AL, Catteruccia F, Church GM. Concerning RNA-guided gene drives for the alteration of wild populations. Elife. 2014;3. 10.7554/eLife.03401 PMC4117217 25035423

[148] Macias VM, Jimenez AJ, Burini-Kojin B, Pledger D, Jasinskiene N, Phong CH (2017). nanos-Driven expression of piggyBac transposase induces mobilization of a synthetic autonomous transposon in the malaria vector mosquito, *Anopheles stephensi*. Insect Biochem Mol Biol.

[149] Krzywinska E, Dennison NJ, Lycett GJ, Krzywinski J (2016). A maleness gene in the malaria mosquito *Anopheles gambiae*. Science.

[150] Franz G, McInnis DO, Morse JG (1995). A promising new twist - a genetic sexing strain based on a temperature sensitive lethal (tsl) mutation. The Mediterranean fruit fly in California: defining critical research.

[151] Jasinskiene N, Coates CJ, Benedict MQ, Cornel AJ, Rafferty CS, James AA, Collins FH (1998). Stable transformation of the yellow fever mosquito, *Aedes aegypti*, with the *Hermes* element from the housefly. Proc Natl Acad Sci U S A.

[152] Allen ML, O'Brochta DA, Atkinson PW, Levesque CS (2001). Stable, germ-line transformation of *Culex quinquefasciatus* (Diptera: Culicidae). J Med Entomol.

[153] Catteruccia F, Nolan T, Blass C, Muller HM, Crisanti A, Kafatos FC, Loukeris TG (2000). Toward *Anopheles* transformation: *Minos* element activity in anopheline cells and embryos. Proc Natl Acad Sci, USA.

[154] Coates CJ, Jasinskiene N, Miyashiro L, James AA (1998). *Mariner* transposition and transformation of the yellow fever mosquito, *Aedes aegypti*. Proc Natl Acad Sci U S A.

[155] Grossman GL, Rafferty CS, Clayton JR, Stevens TK, Mukabayire O, Benedict MQ (2001). Germline transformation of the malaria vector, *Anopheles gambiae*, with the *piggyBac* transposable element. Insect Molecular Biology.

[156] Nolan T, Bower TM, Brown AE, Crisanti A, Catteruccia F (2002). *piggyBac*-mediated germline transformation of the malaria mosquito *Anopheles stephensi* using the red fluorescent protein dsRED as a selectable marker. Journal of Biological Chemistry.

[157] Perera OP, Harrell R, Handler AM (2002). Germ-line transformation of the South American malaria vector, *Anopheles albimanus*, with a *piggyBac*/EGFP transposon vector is routine and highly efficient. Insect Molecular Biology.

[158] Rodrigues FG, Oliveira SB, Rocha BC, Moreira LA (2006). Germline transformation of *Aedes fluviatilis* (Diptera:Culicidae) with the *piggyBac* transposable element. Mem Inst Oswaldo Cruz.

[159] Rowan KH, Orsetti J, Atkinson PW, O'Brochta DA (2004). Tn5 as an insect gene vector. Insect Biochem Mol Biol.

